# Supplementary material for: Megapixel camera arrays enable high-resolution animal tracking in multiwell plates
Source: Commun Biol. 2022 Mar 23;5:253. doi: 10.1038/s42003-022-03206-1 (PMC8943053; doi:10.1038/s42003-022-03206-1)
Supplement: Supplementary file 2 — Supplemental Material pdf [file 42003_2022_3206_MOESM2_ESM.pdf]

Supplementary figures and tables

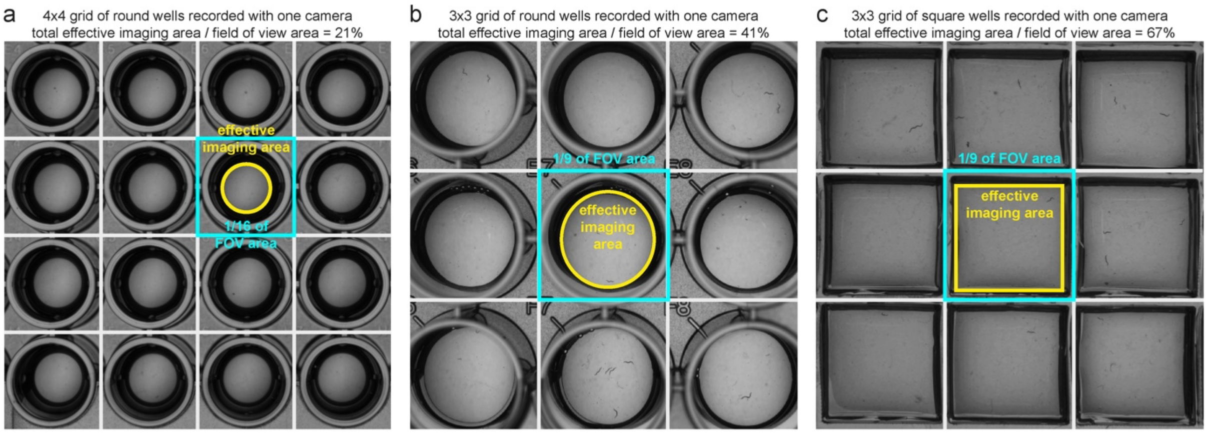

Supplementary Figure 1: Alternative plate designs investigated. **a**) Round wells are less optimal than square wells as a large part of the field of view is lost to the area between wells, thus reducing the effective area for behaviour. Round wells further lose more effective area due to the shadows cast by the separators. **b**) Using larger round wells (here a 48 multiwell plate) yields an increase in the effective area available for imaging, relative to the field of view. Throughput is however negatively affected, as fewer wells are imaged at the same time. **c**) A prototype custom made 54 multiwell plate. Custom plates with shallow wells and thin separators are the best option to maximise effective imaging area, but this comes with increased manufacturing costs and undermines interoperability with established automated liquid handling instruments.

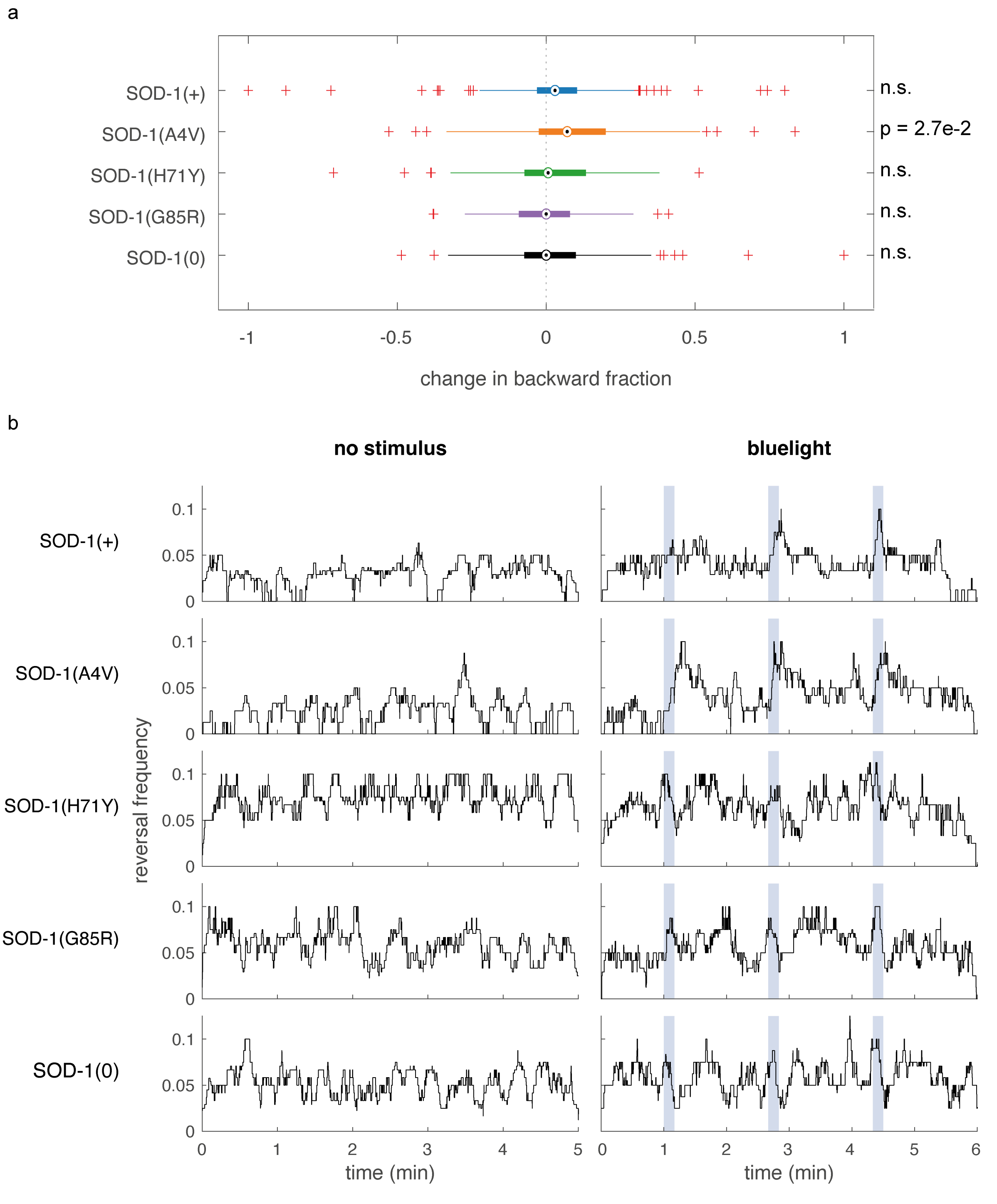

Supplementary Figure 2: Backward locomotion in ALS disease models. a) Changes in the overall fraction of backward locomotion upon blue light stimulation. The difference was calculated by subtracting the average feature values over the t = 50-60 second pre-stimulus window from those over the t = 65-75 second first blue light pulse window (these correspond to the first and the second time points in Figure 3d, respectively. Two sample t-test against SOD-1(+) control strain (n.s. not significant). Boxes show median and 25th and 75th percentiles. **b**) Reversal frequency per worm per second, calculated for each frame at 25 fps. A reversal was detected when a worm changes its motion state from forward or paused to backwards. A 10 second sliding window was used to calculate reversal frequency, then the data was smoothed over a 3 second window. A random sample of 20 independent experiments was used to generate the left and the right column plots for each strain.

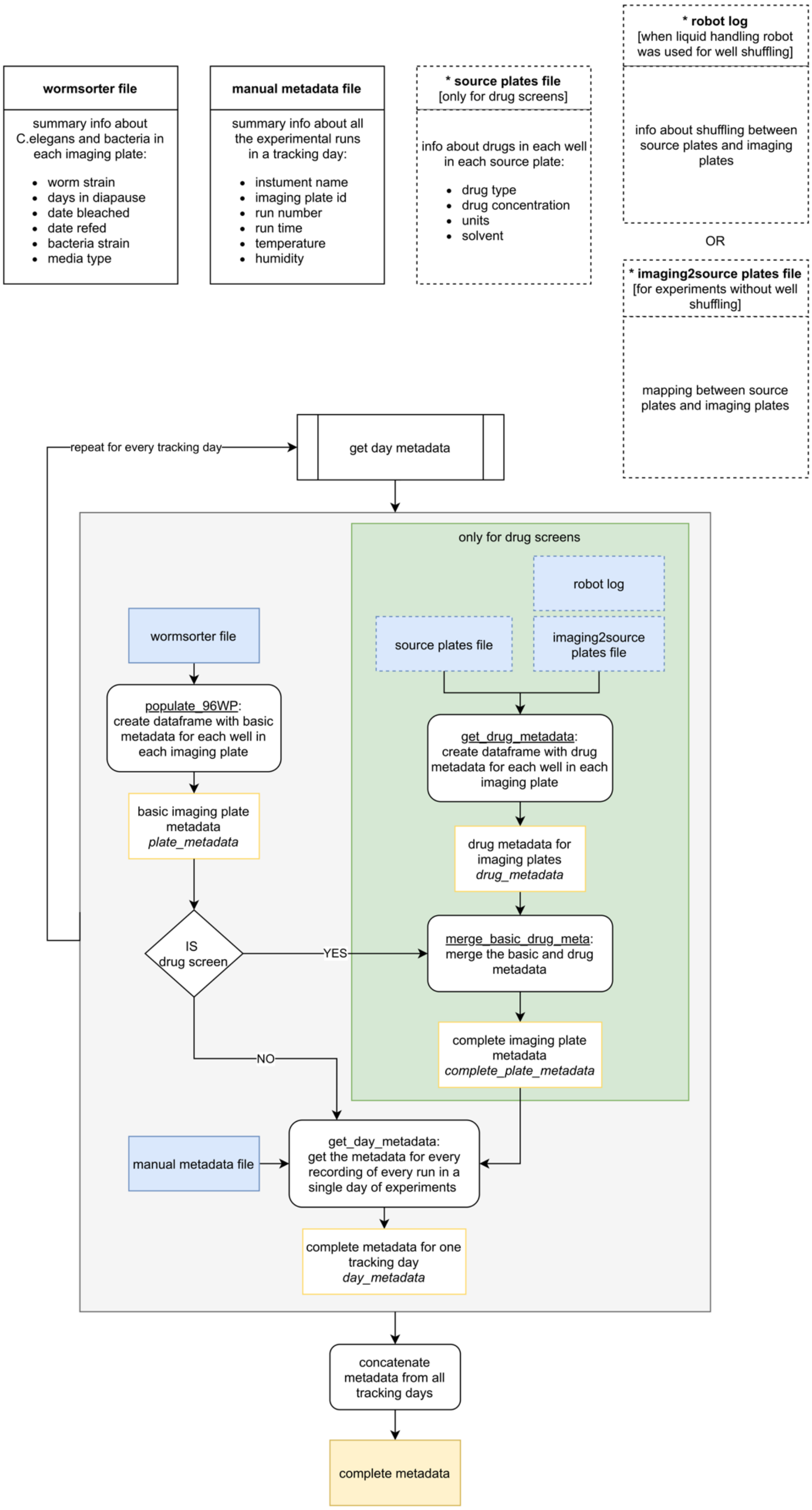

Supplementary Figure 3: Flow chart of the full metadata compilation based on standardised experimental records when imaging with the Kastl - HighRes. As mentioned in the methods section "Data provenance", experiments with multiwell plates pose challenges in terms of keeping track of data provenance. Our pipeline to handle the automatic creation of metadata can flexibly accommodate different experimental needs (objects marked with * and surrounded by dashed boxes are optional and only present in some experimental designs).

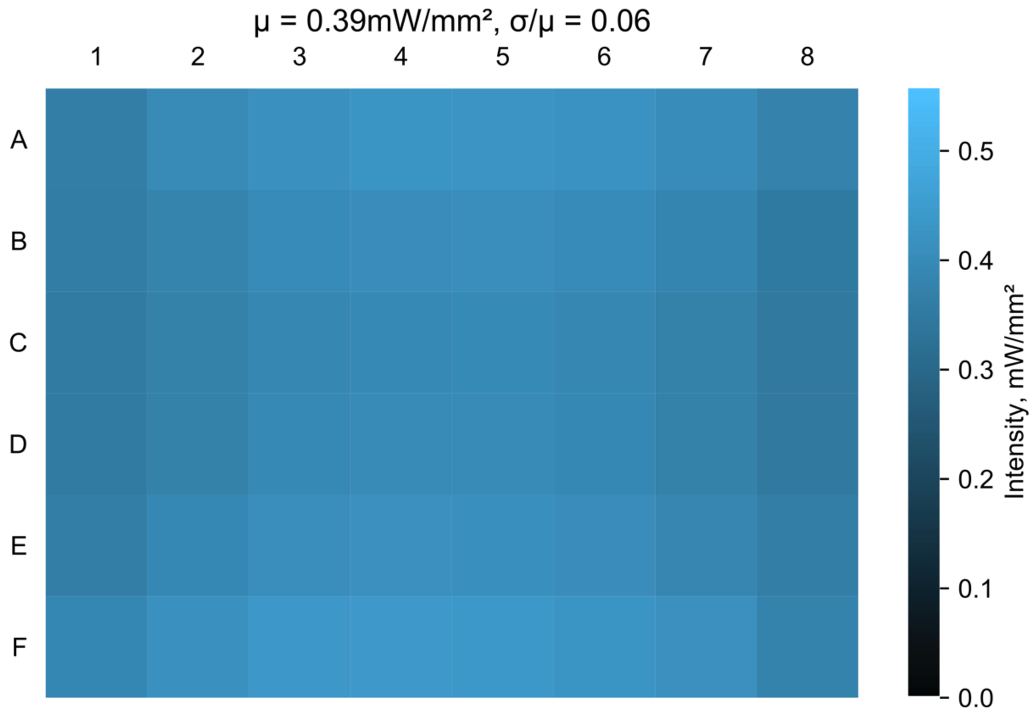

Supplementary Figure 4: Representative heatmap showing the intensity of the blue light emitted by the blue LEDs, built by taking 48 measurements in a 6-by-8 grid spanning the sample area, using a Coherent® Fieldmaster power meter (Coherent Inc, USA).

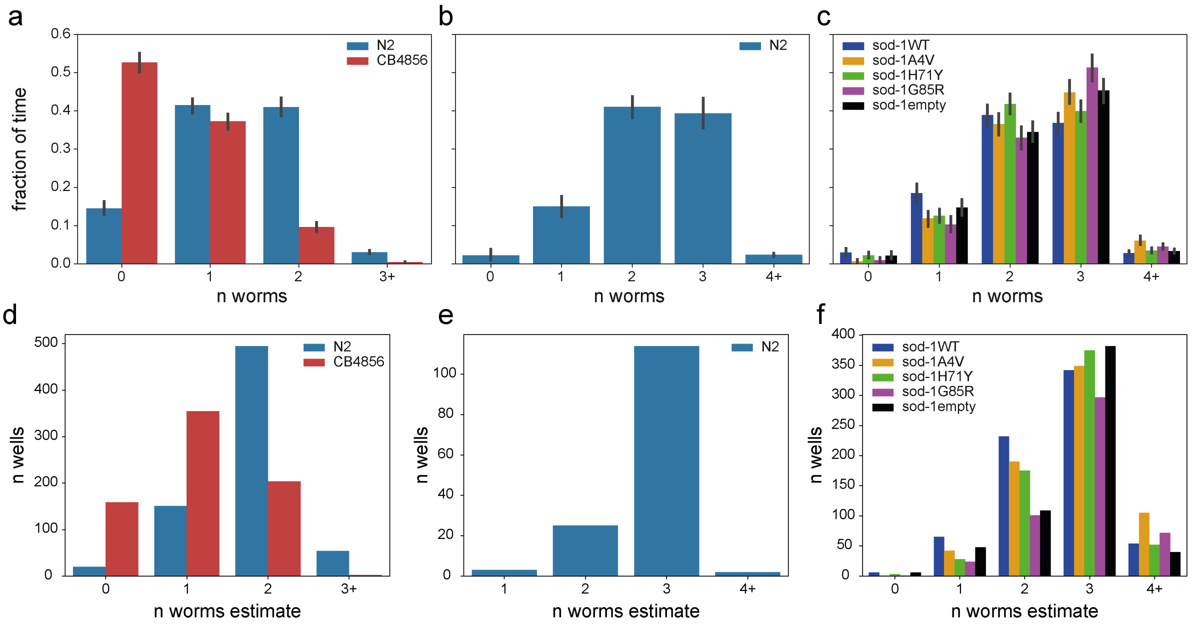

Supplementary Figure 5: Worms can crawl into the shaded area at the edge of the well, preventing perfect tracking of all worms throughout the video. **a**) Fraction of time in which *n* worms are simultaneously tracked, in an experiment with a nominal 2 worms per well. Three worms are occasionally observed either because of loading errors or cases where non-worm objects are not correctly filtered by the CNN worm classifier. **b**, **c**) Dispensing 3 worms per well significantly decreases the fraction of time in which 0 or 1 worms are tracked. Values shown are averages across all wells in each experiment, and error bars show 95% confidence interval via bootstrapping. **d**, **e**, **f**) Number of wells in which *n* worms were imaged simultaneously. The nominal number of worms is 2, 3, and 3 in the three panels respectively.

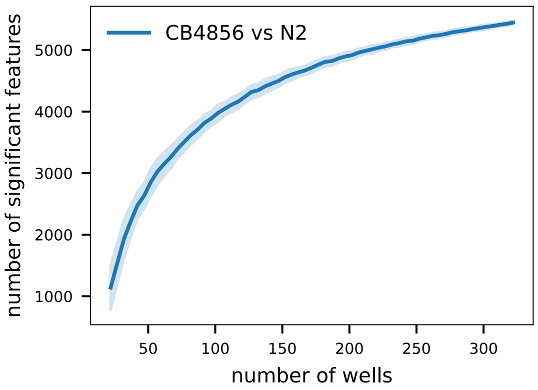

Supplementary Figure 6: The number of features deemed to be significantly different between the Hawaiian strain and the N2 control (Kruskal-Wallis with Benjamin-Yekutieli correction for multiple comparison, false discovery rate 0.05) increases as a function of the number of wells imaged. This figure was obtained by repeatedly sampling an ever-increasing number of wells from the full dataset featured in Figure 3a,b) Solid line is the mean, shaded area is the standard deviation across 100 random samplings without substitution.

Supplementary Table 1: List of strains in this study

Strain name	Genotype
N2	Bristol wild-type reference strain
CB4856	Wild isolate
CX11314	Wild isolate
DL238	Wild isolate
ED3017	Wild isolate
EG4725	Wild isolate
JT11398	Wild isolate
JU258	Wild isolate
JU775	Wild isolate
LKC34	Wild isolate
MY16	Wild isolate
MY23	Wild isolate
HA2427	sod-1(tm776) II; unc-119(ed3) III; rtSi001[sod-1p::sod-1(WT); Cbr-unc-119(+)] IV
HA2464	sod-1(tm776) II; unc-119(+) III; rtSi008 [sod-1p::sod-1A4V^{M} ::sod-1 3'UTR + Cbr-unc-119(+)] IV
HA2425	sod-1(tm776) II; unc-119(+) III; rtSi007 [sod-1p::sod-1H71Y^{M} ::sod-1 3'UTR + Cbr-unc-119(+)] IV
HA2426	sod-1(tm776) II; unc-119(+) III; rtSi006 [sod-1p::sod-1G85R^{M} ::sod-1 3'UTR + Cbr-unc-119(+)] IV
HA2622	sod-1(tm776) II; unc-119(+) III; rtSi026 [Cbr-unc-119(+)] IV

Supplementary Table 2: detailed protocols

Figure panel	Protocol	Number of worms per well	Acclimatisation time (hours)
Figure 2	https://dx.doi.org/10.17504/protocols.io.bn5zmg76	3	4.5
Figure 3a-b	https://dx.doi.org/10.17504/protocols.io.9vqh65w	2	4.5
Figure 3c-e	https://dx.doi.org/10.17504/protocols.io.bsicncaw	3	1.5
Figure 4, Supplementary Figure 2	https://dx.doi.org/10.17504/protocols.io.bsicncaw	3	1.5
Figure 5	https://dx.doi.org/10.17504/protocols.io.bs6znhf6	3	4.5

Note: Acclimatisation time is defined here as time of imaging – (start time on COPAS worm sorting + end time on COPAS worm sorting) / 2.

Supplementary Table 3: Sample size for all experiments involving multiple strains.

Figure	worm strain	n wells	across n plates	across n days	worms per well
Fig 2	N2	34	32	3	3
Fig 2	JT11398	21	19	3	3
Fig 2	MY16	27	24	3	3
Fig 2	EG4725	29	26	3	3
Fig 2	JU258	25	23	3	3
Fig 2	JU775	25	21	4	3
Fig 2	LKC34	27	26	3	3
Fig 2	CB4856	29	27	4	3
Fig 2	DL238	16	15	3	3
Fig 2	ED3017	20	20	3	3
Fig 2	MY23	23	22	3	3
Fig 3a (left)	N2	377	149	6	2
Fig 3a (left)	CB4856	115	82	6	2
Fig 3a (right)	N2	398	152	6	2
Fig 3a (right)	CB4856	98	73	6	2
Fig 3b	N2	529	155	6	2
Fig 3b	CB4856	396	152	6	2
Fig 3c-e	N2	144	4	4	3
Fig 4, S2	sod-1(+)	232	15	3	3
Fig 4, S2	sod-1(A4V)	228	15	3	3
Fig 4, S2	sod-1(H71Y)	211	15	3	3
Fig 4, S2	sod-1(G85R)	165	13	3	3
Fig 4, S2	sod-1(0)	195	13	3	3

Supplementary Table 4: Sample size for each compound and concentration featured in Figure 5. Three N2 worms were dispensed in each well.

compound and concentration (μM)	wells	plates	days	compound and concentration (μM)	wells	plates	days
Adenosine 5'-monophosphate monohydrate_1.0	8	8	3	Labetalol hydrochloride_1.0	6	6	3
Adenosine 5'-monophosphate monohydrate_10.0	8	8	3	Labetalol hydrochloride_10.0	6	6	3
Adenosine 5'-monophosphate monohydrate_100.0	8	8	3	Labetalol hydrochloride_100.0	7	7	4
Adiphenine hydrochloride_1.0	7	7	3	Laudanosine (R,S)_1.0	7	7	3
Adiphenine hydrochloride_10.0	7	7	3	Laudanosine (R,S)_10.0	7	7	3
Adiphenine hydrochloride_100.0	7	7	3	Laudanosine (R,S)_100.0	6	6	3
Agmatine sulfate_0.25	5	5	2	Lomefloxacin hydrochloride_0.5	4	4	2
Agmatine sulfate_2.5	8	8	3	Lomefloxacin hydrochloride_5.0	2	2	1
Agmatine sulfate_25.0	7	7	3	Lomefloxacin hydrochloride_50.0	3	3	2
Alverine citrate salt_1.0	8	8	3	Loperamide hydrochloride_1.0	7	7	3
Alverine citrate salt_10.0	8	8	3	Loperamide hydrochloride_10.0	6	6	2
Alverine citrate salt_100.0	7	7	3	Loperamide hydrochloride_100.0	7	7	3
Ambroxol hydrochloride_1.0	7	7	3	Mebeverine hydrochloride_1.0	5	5	3
Ambroxol hydrochloride_10.0	7	7	3	Mebeverine hydrochloride_10.0	6	6	3
Ambroxol hydrochloride_100.0	7	7	3	Mebeverine hydrochloride_100.0	8	8	3
Amikacin_1.0	6	6	3	Meclofenamic acid sodium salt monohydrate_1.0	4	4	2
Amikacin_10.0	7	7	2	Meclofenamic acid sodium salt monohydrate_10.0	7	7	3
Amikacin_100.0	7	7	3	Meclofenamic acid sodium salt monohydrate_100.0	6	6	3
Amiodarone hydrochloride_0.5	8	8	3	Melatonin_1.0	7	7	3
Amiodarone hydrochloride_5.0	7	7	3	Melatonin_10.0	6	6	3
Amiodarone hydrochloride_50.0	5	5	3	Melatonin_100.0	7	7	3
Amoxicillin_0.5	7	7	2	Mepenzolate bromide_1.0	6	6	3
Amoxicillin_5.0	8	8	3	Mepenzolate bromide_10.0	8	8	3
Amoxicillin_50.0	6	6	3	Mepenzolate bromide_100.0	6	6	3
Ampicillin trihydrate_1.0	7	7	3	Mesoridazine besylate_1.0	6	6	2
Ampicillin trihydrate_10.0	5	5	2	Mesoridazine besylate_10.0	7	7	3
Ampicillin trihydrate_100.0	3	3	2	Mesoridazine besylate_100.0	7	7	3
Antazoline hydrochloride_1.0	6	6	3	Metaraminol bitartrate_1.0	7	7	2
Antazoline hydrochloride_10.0	7	7	4	Metaraminol bitartrate_10.0	6	6	2
Antazoline hydrochloride_100.0	6	6	3	Metaraminol bitartrate_100.0	8	8	3
Arecoline hydrobromide_1.0	8	8	3	Metformin hydrochloride_1.0	8	8	3
Arecoline hydrobromide_10.0	7	7	2	Metformin hydrochloride_10.0	6	6	3
Arecoline hydrobromide_100.0	7	7	3	Metformin hydrochloride_100.0	7	7	3
Ascorbic acid_1.0	6	6	3	Methapyrilene hydrochloride_1.0	5	5	3
Ascorbic acid_10.0	7	7	3	Methapyrilene hydrochloride_10.0	6	6	3
Ascorbic acid_100.0	7	7	3	Methapyrilene hydrochloride_100.0	6	6	3
Atropine sulfate monohydrate_1.0	7	7	3	Methocarbamol_1.0	7	7	3
Atropine sulfate monohydrate_10.0	4	4	3	Methocarbamol_10.0	7	7	3
Atropine sulfate monohydrate_100.0	6	6	3	Methocarbamol_100.0	7	7	3
Bacampicillin hydrochloride_1.0	7	7	3	Metoprolol-(+,-) (+)-tartrate salt_1.0	7	7	3
Bacampicillin hydrochloride_10.0	6	6	2	Metoprolol-(+,-) (+)-tartrate salt_10.0	8	8	3
Bacampicillin hydrochloride_100.0	7	7	3	Metoprolol-(+,-) (+)-tartrate salt_100.0	6	6	3
Benfluorex hydrochloride_1.0	8	8	3	Metronidazole_1.0	8	4	3
Benfluorex hydrochloride_10.0	7	7	3	Metronidazole_10.0	8	4	3
Benfluorex hydrochloride_100.0	5	5	3	Metronidazole_100.0	6	3	2
Benoxinate hydrochloride_1.0	7	7	3	Mexiletine hydrochloride_1.0	5	5	3
Benoxinate hydrochloride_10.0	5	5	3	Mexiletine hydrochloride_10.0	7	7	4
Benoxinate hydrochloride_100.0	7	7	3	Mexiletine hydrochloride_100.0	4	4	3
Benzydamine hydrochloride_1.0	6	6	3	Midodrine hydrochloride_1.0	7	7	2
Benzydamine hydrochloride_10.0	7	7	3	Midodrine hydrochloride_10.0	7	7	2
Benzydamine hydrochloride_100.0	8	8	3	Midodrine hydrochloride_100.0	6	6	2
Brompheniramine maleate_1.0	5	5	2	Minoxidil_0.25	8	8	3
Brompheniramine maleate_10.0	7	7	3	Minoxidil_2.5	7	7	2
Brompheniramine maleate_100.0	4	4	2	Minoxidil_25.0	8	8	3
Buflomedil hydrochloride_1.0	8	8	3	Molsidomine_1.0	7	7	3
Buflomedil hydrochloride_10.0	8	8	3	Molsidomine_10.0	7	7	3
Buflomedil hydrochloride_100.0	8	8	3	Molsidomine_100.0	5	5	3

compound and concentration (µM)	wells	plates	days	compound and concentration (µM)	wells	plates	days
Bupivacaine hydrochloride _1.0	7	7	3	Nalidixic acid sodium salt hydrate_0.25	6	6	2
Bupivacaine hydrochloride _10.0	8	8	3	Nalidixic acid sodium salt hydrate_2.5	7	7	3
Bupivacaine hydrochloride _100.0	5	5	3	Nalidixic acid sodium salt hydrate_25.0	5	5	2
Buspirone hydrochloride_0.5	8	8	3	Naphazoline hydrochloride_0.5	7	7	3
Buspirone hydrochloride_5.0	8	8	3	Naphazoline hydrochloride_5.0	5	5	3
Buspirone hydrochloride_50.0	8	8	3	Naphazoline hydrochloride_50.0	7	7	3
CSAA466656_1.0	3	2	2	Nefopam hydrochloride_1.0	6	6	4
CSAA466656_10.0	1	1	1	Nefopam hydrochloride_10.0	6	6	4
CSAA466656_100.0	3	3	3	Nefopam hydrochloride_100.0	6	6	4
Captopril_1.0	7	7	3	Neostigmine bromide_1.0	6	6	3
Captopril_10.0	6	6	2	Neostigmine bromide_10.0	6	6	2
Captopril_100.0	7	7	3	Neostigmine bromide_100.0	6	6	2
Carbenicillin disodium salt_1.0	7	7	3	Nitrofurantoin_1.0	7	7	3
Carbenicillin disodium salt_10.0	6	6	2	Nitrofurantoin_10.0	7	7	3
Carbenicillin disodium salt_100.0	6	6	3	Nitrofurantoin_100.0	7	7	3
Cefadroxil_0.5	8	8	3	Nortriptyline hydrochloride_1.0	6	6	3
Cefadroxil_5.0	7	7	3	Nortriptyline hydrochloride_10.0	5	5	2
Cefadroxil_50.0	8	8	3	Nortriptyline hydrochloride_100.0	5	5	2
Chenodiol_1.0	7	7	3	Olanzapine_1.0	7	4	3
Chenodiol_10.0	7	7	3	Olanzapine_10.0	7	4	3
Chenodiol_100.0	7	7	3	Olanzapine_100.0	7	4	3
Chloroquine diphosphate_0.5	6	6	3	Ornidazole_1.0	7	7	3
Chloroquine diphosphate_5.0	7	7	3	Ornidazole_10.0	7	7	3
Chloroquine diphosphate_50.0	7	7	3	Ornidazole_100.0	7	7	3
Chlorpheniramine maleate_1.0	7	7	3	Orphenadrine hydrochloride_1.0	5	5	2
Chlorpheniramine maleate_10.0	7	7	3	Orphenadrine hydrochloride_10.0	5	5	3
Chlorpheniramine maleate_100.0	7	7	3	Orphenadrine hydrochloride_100.0	4	4	2
Chlorpromazine_1.0	7	4	3	Oxethazaine_1.0	7	7	3
Chlorpromazine_10.0	8	4	3	Oxethazaine_10.0	6	6	3
Chlorpromazine_100.0	3	2	2	Oxethazaine_100.0	7	7	3
Chlorzoxazone_1.0	6	6	3	Oxolinic acid_0.25	6	6	2
Chlorzoxazone_10.0	7	7	3	Oxolinic acid_2.5	7	7	2
Chlorzoxazone_100.0	7	7	3	Oxolinic acid_25.0	5	5	2
Ciclopirox ethanolamine_1.0	7	7	3	Oxymetazoline hydrochloride_1.0	4	4	3
Ciclopirox ethanolamine_10.0	5	5	2	Oxymetazoline hydrochloride_10.0	6	6	4
Ciclopirox ethanolamine_100.0	5	5	2	Oxymetazoline hydrochloride_100.0	6	6	4
Cinoxacin_0.5	7	4	3	Pentoxifylline_1.0	7	7	3
Cinoxacin_5.0	4	3	2	Pentoxifylline_10.0	7	7	2
Cinoxacin_50.0	6	3	2	Pentoxifylline_100.0	7	7	2
Clarithromycin_0.5	6	6	2	Phenacetin_1.0	8	8	3
Clarithromycin_5.0	7	7	3	Phenacetin_10.0	8	8	3
Clarithromycin_50.0	6	6	3	Phenacetin_100.0	6	6	3
Clomiphene citrate (Z,E)_1.0	6	6	2	Pheniramine maleate_1.0	8	8	3
Clomiphene citrate (Z,E)_10.0	8	8	3	Pheniramine maleate_10.0	6	6	3
Clomiphene citrate (Z,E)_100.0	5	5	3	Pheniramine maleate_100.0	5	5	3
Colistin sulfate_0.5	4	4	2	Phenylpropanolamine hydrochloride_1.0	8	8	3
Colistin sulfate_5.0	6	6	2	Phenylpropanolamine hydrochloride_10.0	8	8	3
Colistin sulfate_50.0	5	5	2	Phenylpropanolamine hydrochloride_100.0	8	8	3
Convolamine hydrochloride_0.5	6	6	3	Pimozide_0.25	6	6	3
Convolamine hydrochloride_5.0	6	6	3	Pimozide_2.5	7	7	3
Convolamine hydrochloride_50.0	5	5	2	Pimozide_25.0	7	7	3
Dehydrocholic acid_1.0	7	7	3	Pipenzolate bromide_1.0	6	6	3
Dehydrocholic acid_10.0	6	6	2	Pipenzolate bromide_10.0	7	7	3
Dehydrocholic acid_100.0	7	7	3	Pipenzolate bromide_100.0	7	7	3
Dextromethorphan hydrobromide monohydrate_1.0	7	7	3	Piracetam_1.0	7	7	3
Dextromethorphan hydrobromide monohydrate_10.0	7	7	2	Piracetam_10.0	7	7	3
Dextromethorphan hydrobromide monohydrate_100.0	8	8	3	Piracetam_100.0	8	8	3
Dicloxacillin sodium salt hydrate_1.0	7	7	3	Praziquantel_1.0	7	7	4
Dicloxacillin sodium salt hydrate_10.0	7	7	3	Praziquantel_10.0	7	7	4
Dicloxacillin sodium salt hydrate_100.0	7	7	3	Praziquantel_100.0	7	7	4
Diethylcarbamazine citrate_1.0	7	7	3	Prednisone_1.0	7	7	3

compound and concentration (µM)	wells	plates	days	compound and concentration (µM)	wells	plates	days
Diethylcarbamazine citrate_10.0	8	8	3	Prednisone_10.0	7	7	3
Diethylcarbamazine citrate_100.0	8	8	3	Prednisone_100.0	7	7	3
Dihydrostreptomycin sulfate_0.5	7	7	3	Prilocaine hydrochloride_1.0	8	8	3
Dihydrostreptomycin sulfate_5.0	6	6	3	Prilocaine hydrochloride_10.0	7	7	3
Dihydrostreptomycin sulfate_50.0	5	5	3	Prilocaine hydrochloride_100.0	7	7	2
Diltiazem hydrochloride_1.0	7	7	3	Probenecid_1.0	7	7	3
Diltiazem hydrochloride_10.0	7	7	3	Probenecid_10.0	6	6	3
Diltiazem hydrochloride_100.0	6	6	2	Probenecid_100.0	5	5	3
Diphenhydramine hydrochloride_1.0	6	6	3	Procaine hydrochloride_1.0	8	8	3
Diphenhydramine hydrochloride_10.0	7	7	3	Procaine hydrochloride_10.0	8	8	3
Diphenhydramine hydrochloride_100.0	7	7	3	Procaine hydrochloride_100.0	7	7	2
Diprophylline_1.0	8	8	3	Proglumide_1.0	6	6	3
Diprophylline_10.0	5	5	3	Proglumide_10.0	6	6	3
Diprophylline_100.0	7	7	3	Proglumide_100.0	6	6	3
Disopyramide_1.0	7	7	4	Propafenone hydrochloride_1.0	7	7	3
Disopyramide_10.0	6	6	3	Propafenone hydrochloride_10.0	7	7	3
Disopyramide_100.0	5	5	2	Propafenone hydrochloride_100.0	4	4	3
Doxylamine succinate_1.0	6	6	4	Pyrazinamide_1.0	7	7	3
Doxylamine succinate_10.0	6	6	3	Pyrazinamide_10.0	7	7	3
Doxylamine succinate_100.0	4	4	2	Pyrazinamide_100.0	7	7	3
Droperidol_1.0	8	8	3	Pyrilamine maleate_1.0	8	8	3
Droperidol_10.0	8	8	3	Pyrilamine maleate_10.0	8	8	3
Droperidol_100.0	8	8	3	Pyrilamine maleate_100.0	8	8	3
Dropropizine (R,S)_1.0	5	5	2	Pyrithione sodium salt_1.0	7	7	3
Dropropizine (R,S)_10.0	6	6	3	Pyrithione sodium salt_10.0	7	7	3
Dropropizine (R,S)_100.0	5	5	2	Pyrithione sodium salt_100.0	7	7	3
Dyclonine hydrochloride_0.5	6	6	4	Scopolamin-N-oxide hydrobromide_1.0	5	5	3
Dyclonine hydrochloride_5.0	7	7	4	Scopolamin-N-oxide hydrobromide_10.0	5	5	3
Dyclonine hydrochloride_50.0	7	7	4	Scopolamin-N-oxide hydrobromide_100.0	6	6	4
Epiandrosterone_1.0	6	6	2	Strophantine octahydrate_1.0	6	6	2
Epiandrosterone_10.0	5	5	2	Strophantine octahydrate_10.0	6	6	2
Epiandrosterone_100.0	6	6	3	Strophantine octahydrate_100.0	5	5	2
Estradiol-17 beta_1.0	6	6	3	Sulfadiazine_1.0	6	6	4
Estradiol-17 beta_10.0	5	5	2	Sulfadiazine_10.0	6	6	4
Estradiol-17 beta_100.0	5	5	2	Sulfadiazine_100.0	5	5	3
Fenbufen_1.0	7	7	2	Sulfaguanidine_1.0	7	7	3
Fenbufen_10.0	7	7	3	Sulfaguanidine_10.0	6	6	2
Fenbufen_100.0	7	7	2	Sulfaguanidine_100.0	7	7	3
Fenoterol hydrobromide_1.0	7	7	3	Sulfathiazole_1.0	7	7	3
Fenoterol hydrobromide_10.0	7	7	3	Sulfathiazole_10.0	7	7	3
Fenoterol hydrobromide_100.0	8	8	3	Sulfathiazole_100.0	7	7	3
Flavoxate hydrochloride_0.5	6	6	3	Sulfisoxazole_1.0	5	5	2
Flavoxate hydrochloride_5.0	5	5	2	Sulfisoxazole_10.0	5	5	2
Flavoxate hydrochloride_50.0	7	7	4	Sulfisoxazole_100.0	5	5	2
Flufenamic acid_1.0	6	6	3	Suxibuzone_1.0	8	8	3
Flufenamic acid_10.0	7	7	3	Suxibuzone_10.0	8	8	3
Flufenamic acid_100.0	8	8	3	Suxibuzone_100.0	7	7	3
Flunarizine dihydrochloride_0.25	6	6	3	Tamoxifen citrate_1.0	7	7	3
Flunarizine dihydrochloride_2.5	8	8	3	Tamoxifen citrate_10.0	7	7	3
Flunarizine dihydrochloride_25.0	5	5	2	Tamoxifen citrate_100.0	6	6	3
Fusaric acid_1.0	6	6	2	Thiamphenicol_1.0	3	3	2
Fusaric acid_10.0	6	6	2	Thiamphenicol_10.0	6	6	3
Fusaric acid_100.0	6	6	2	Thiamphenicol_100.0	3	3	1
Gabazine bromide_1.0	6	6	2	Thyroxine (L)_1.0	8	8	3
Gabazine bromide_10.0	6	6	3	Thyroxine (L)_10.0	8	8	3
Gabazine bromide_100.0	5	5	2	Thyroxine (L)_100.0	8	8	3
Glibenclamide_1.0	8	8	3	Todralazine hydrochloride_1.0	7	7	3
Glibenclamide_10.0	6	6	3	Todralazine hydrochloride_10.0	8	8	3
Glibenclamide_100.0	5	5	2	Todralazine hydrochloride_100.0	6	6	2
Gramine_1.0	5	5	2	Tolazoline hydrochloride_1.0	5	5	2
Gramine_10.0	6	6	3	Tolazoline hydrochloride_10.0	6	6	3
Gramine_100.0	5	5	3	Tolazoline hydrochloride_100.0	5	5	3
Guaifenesin_1.0	8	8	3	Tolfenamic acid_1.0	6	6	3
Guaifenesin_10.0	7	7	3	Tolfenamic acid_10.0	7	7	3
Guaifenesin_100.0	8	8	3	Tolfenamic acid_100.0	7	7	2

compound and concentration (μM)	wells	plates	days	compound and concentration (μM)	wells	plates	days
Guanethidine sulfate _0.25	5	5	3	Tranexamic acid_0.5	7	7	3
Guanethidine sulfate _2.5	5	5	3	Tranexamic acid_5.0	7	7	3
Guanethidine sulfate _25.0	8	8	3	Tranexamic acid_50.0	7	7	3
Haloperidol_1.0	7	4	3	Trazodone hydrochloride_0.5	8	8	3
Haloperidol_10.0	8	4	3	Trazodone hydrochloride_5.0	7	7	3
Haloperidol_100.0	7	4	3	Trazodone hydrochloride_50.0	8	8	3
Hemicholinium bromide_1.0	7	7	3	Trigonelline hydrochloride_0.5	6	6	3
Hemicholinium bromide_10.0	7	7	3	Trigonelline hydrochloride_5.0	7	7	3
Hemicholinium bromide_100.0	6	6	2	Trigonelline hydrochloride_50.0	7	7	3
Homatropine hydrobromide (R,S)_1.0	7	7	3	Trihexyphenidyl-D,L Hydrochloride_0.5	7	7	3
Homatropine hydrobromide (R,S)_10.0	6	6	3	Trihexyphenidyl-D,L Hydrochloride_5.0	7	7	3
Homatropine hydrobromide (R,S)_100.0	7	7	3	Trihexyphenidyl-D,L Hydrochloride_50.0	8	8	3
Homochlorcyclizine dihydrochloride_1.0	6	6	3	Trimethadione_1.0	7	7	3
Homochlorcyclizine dihydrochloride_10.0	8	8	3	Trimethadione_10.0	7	7	3
Homochlorcyclizine dihydrochloride_100.0	2	2	1	Trimethadione_100.0	6	6	3
Hydroquinine hydrobromide hydrate_1.0	8	8	3	Triprolidine hydrochloride_1.0	6	6	3
Hydroquinine hydrobromide hydrate_10.0	7	7	3	Triprolidine hydrochloride_10.0	7	7	4
Hydroquinine hydrobromide hydrate_100.0	8	8	3	Triprolidine hydrochloride_100.0	7	7	4
Hydroxyzine dihydrochloride_1.0	6	6	2	Troleandomycin_1.0	7	7	2
Hydroxyzine dihydrochloride_10.0	6	6	3	Troleandomycin_10.0	7	7	2
Hydroxyzine dihydrochloride_100.0	6	6	3	Troleandomycin_100.0	4	4	2
Isoxsuprine hydrochloride_1.0	6	6	3	Valsartan_1.0	8	8	3
Isoxsuprine hydrochloride_10.0	7	7	3	Valsartan_10.0	8	8	3
Isoxsuprine hydrochloride_100.0	7	7	3	Valsartan_100.0	8	8	3
Kanamycin A sulfate_0.5	8	8	3	Verapamyl hydrochloride_1.0	6	6	3
Kanamycin A sulfate_5.0	6	6	3	Verapamyl hydrochloride_10.0	7	7	3
Kanamycin A sulfate_50.0	7	7	2	Verapamyl hydrochloride_100.0	7	7	3
Ketoprofen_1.0	8	8	3	Xylometazoline hydrochloride_1.0	8	8	3
Ketoprofen_10.0	8	8	3	Xylometazoline hydrochloride_10.0	8	8	3
Ketoprofen_100.0	8	8	3	Xylometazoline hydrochloride_100.0	7	7	2
DMSO_0	50	36	4	Yohimbine hydrochloride_0.5	7	7	3
DMSO_1	50	34	4	Yohimbine hydrochloride_5.0	7	7	3
DMSO_2	49	36	4	Yohimbine hydrochloride_50.0	7	7	3
DMSO_3	49	35	4	DMSO_5	49	37	4
DMSO_4	49	36	4				

Note: the numerous control data points (DMSO) were randomly partitioned into 6 groups using scikit-learn's StratifiedKFold to maintain the correct imaging day distribution.
