# Supplementary material for: Megapixel camera arrays enable high-resolution animal tracking in multiwell plates
Source: Commun Biol. 2022 Mar 23;5:253. doi: 10.1038/s42003-022-03206-1 (PMC8943053; doi:10.1038/s42003-022-03206-1)
Supplement: Supplementary file 3 — Reporting Summary [file 42003_2022_3206_MOESM3_ESM.pdf]

nature portfolio

Corresponding author(s): André Brown

Last updated by author(s): 2022/01/20

Reporting Summary

Nature Portfolio wishes to improve the reproducibility of the work that we publish. This form provides structure for consistency and transparency in reporting. For further information on Nature Portfolio policies, see our Editorial Policies and the Editorial Policy Checklist.

Statistics

For all statistical analyses, confirm that the following items are present in the figure legend, table legend, main text, or Methods section.

n/a	Confirmed	
☐	☒	The exact sample size (n) for each experimental group/condition, given as a discrete number and unit of measurement
☐	☒	A statement on whether measurements were taken from distinct samples or whether the same sample was measured repeatedly
☐	☒	The statistical test(s) used AND whether they are one- or two-sided<br>*Only common tests should be described solely by name; describe more complex techniques in the Methods section.*
☐	☒	A description of all covariates tested
☐	☒	A description of any assumptions or corrections, such as tests of normality and adjustment for multiple comparisons
☐	☒	A full description of the statistical parameters including central tendency (e.g. means) or other basic estimates (e.g. regression coefficient) AND variation (e.g. standard deviation) or associated estimates of uncertainty (e.g. confidence intervals)
☐	☒	For null hypothesis testing, the test statistic (e.g. F, t, r) with confidence intervals, effect sizes, degrees of freedom and P value noted<br>*Give P values as exact values whenever suitable.*
☒	☐	For Bayesian analysis, information on the choice of priors and Markov chain Monte Carlo settings
☒	☐	For hierarchical and complex designs, identification of the appropriate level for tests and full reporting of outcomes
☒	☐	Estimates of effect sizes (e.g. Cohen's d, Pearson's r), indicating how they were calculated

Our web collection on statistics for biologists contains articles on many of the points above.

Software and code

Policy information about availability of computer code

Data collection	Data capture: Motif version 5.1.0, from LoopBio<br>Video storage: https://github.com/loopbio/imgstore<br>Data aquisition: https://github.com/loopbio/python-motifapi
Data analysis	Segmentation and feature extraction: https://github.com/Tierpsy/tierpsy-tracker<br>General tools for statistical tests and classification: https://github.com/Tierpsy/tierpsy-tools-python

For manuscripts utilizing custom algorithms or software that are central to the research but not yet described in published literature, software must be made available to editors and reviewers. We strongly encourage code deposition in a community repository (e.g. GitHub). See the Nature Portfolio guidelines for submitting code & software for further information.

Data

Policy information about availability of data

All manuscripts must include a data availability statement. This statement should provide the following information, where applicable:

- Accession codes, unique identifiers, or web links for publicly available datasets
- A description of any restrictions on data availability
- For clinical datasets or third party data, please ensure that the statement adheres to our policy

The datasets and computer code produced in this study are available in the following databases:
• Tierpsy features, tracking data, and metadata: Zenodo (https://doi.org/10.5281/zenodo.5121521)
• General tools used for analysis and statistical tests: GitHub (github.com/Tierpsy/tierpsy-tools-python)

Field-specific reporting

Please select the one below that is the best fit for your research. If you are not sure, read the appropriate sections before making your selection.

☒ Life sciences ☐ Behavioural & social sciences ☐ Ecological, evolutionary & environmental sciences

For a reference copy of the document with all sections, see nature.com/documents/nr-reporting-summary-flat.pdf

Life sciences study design

All studies must disclose on these points even when the disclosure is negative.

Sample size	No sample size calculations were performed. Based on previous work, three replicates (independent experimental days) with multiple wells recorded per day sufficed to detect the effect of neuroactive compounds on behaviour (McDermott-Rouse et al. (2021) Mol Sys Biol).
Data exclusions	Wells were excluded from analysis by manual inspection. Reasons for exclusion include cracked agar, wet plates (worms swimming rather than crawling), or in drug experiments, precipitated compounds. Wells with too few properly tracked frames were also excluded.
Replication	All experiments were repeated on at least three different days. No further attempts at replication were made.
Randomization	Arrangement of drug treatments within the imaging plates was randomised using a liquid handling robot.<br>In the experiments featured in figure 2, nematodes of different strains were either imaged on different plates, or their positioning within the imaging plate was changed across the experimental days.<br>In the experiments in figure 3A,B and 4, the positioning of strains in the imaging plates was changed across imaging plates and/or imaging days.
Blinding	Blinding was not necessary because the segmentation and feature extraction steps do not depend on user inputs that could bias results. The data exclusion step (marking bad wells) is done manually and is approximately blinded because the user interface serves single wells at a time without annotation and the video-level filenames are date-time strings that are not directly informative of sample conditions.

Reporting for specific materials, systems and methods

We require information from authors about some types of materials, experimental systems and methods used in many studies. Here, indicate whether each material, system or method listed is relevant to your study. If you are not sure if a list item applies to your research, read the appropriate section before selecting a response.

Materials & experimental systems

n/a	Involved in the study	
☒	☐	Antibodies
☒	☐	Eukaryotic cell lines
☒	☐	Palaeontology and archaeology
☐	☒	Animals and other organisms
☒	☐	Human research participants
☒	☐	Clinical data
☒	☐	Dual use research of concern

Methods

n/a	Involved in the study	
☒	☐	ChIP-seq
☒	☐	Flow cytometry
☒	☐	MRI-based neuroimaging

Animals and other organisms

Policy information about studies involving animals; ARRIVE guidelines recommended for reporting animal research

Laboratory animals	Experiments were performed on C. elegans. Specific strains are detailed for each experiment in the manuscript.
Wild animals	The wild isolates in this study are available in strain collections and were not collected as part of this study.
Field-collected samples	This study did not involve field-collected samples.
Ethics oversight	Ethics oversight is not required for work with nematodes.

Note that full information on the approval of the study protocol must also be provided in the manuscript.
